# Peste des petits ruminants pathogenesis on experimental infected goats by the Moroccan 2015 isolate

**DOI:** 10.1186/s12917-019-2164-6

**Published:** 2019-12-16

**Authors:** Z. Bamouh, F. Fakri, M. Jazouli, N. Safini, K. Omari Tadlaoui, M. Elharrak

**Affiliations:** Research and Development, MCI Santé Animale, Lot. 157, Z. I., Sud-Ouest (ERAC) B.P: 278, 28810 Mohammedia, Morocco

**Keywords:** PPR virus, Lineage IV, Experimental infection, Tissue tropism, Pathogenesis, Goats

## Abstract

**Background:**

Peste des petits ruminants (PPR) is a viral disease of major economic importance on small ruminants. Goats are usually known to be more susceptible to the disease. Infection chronology, virus circulation, and the disease early detection need to be better understood. This study evaluates the tissue tropism and pathogenesis of PPR following experimental infection of goats using a lineage IV virus, the most dominant in the world originated from Asia. PPRV infection was experimentally induced in 4 six-month-old goats by intra-nasal and intravenous route of cell virus suspension and from infectious mashed tissue. The clinical signs were observed and goats were euthanized at predetermined clinical score level for post-mortem examinations and PPRV detection by RT-PCR. Clinical signs of infection were present, pyrexia, serous-mucopurulent nasal discharges, coughing, diarrhea and asthenia, for both cell virus suspension and infectious mashed tissue. PPRV genome was highly detected in swabs and tissues with clinical signs dominated by pulmonary attack and digestive symptoms secondary.

**Results:**

Results of this study indicates that PPRV is an invasive infection in animals that in a short period, less than 10 days, invade all vital organs. On live animals, early diagnostic may be easily done on lacrimal and rectal swabs.

**Conclusion:**

The experimental PPRV-infection model using the cell virus suspension is suitable for vaccine evaluation as a standard model.

## Background

Peste des petits ruminants (PPR) represents one of the most economically important disease and imposes a significant constraint on small ruminants [1, 2] and it was considered to be an emerging viral disease of small ruminants caused by a Morbillivirus. PPR is widespread in Africa, Middle East and Southern Asia [3–5].

The PPRV contaminates animals through their oral and nasal passages. After entering into the organism, the initial virus replication occurs within the nasopharyngeal/respiratory epithelium [6, 7], prior to infection of regional lymphoid organs, where a second round of replication occurs [8, 9] and these disseminated the infection to distant organs. The morbilliviruses display a strong tropism for lymphoid tissues and during infection destruction of leucocytes often causes a profound immunosuppression [10].

PPR virus (PPRV) causes severe clinical signs in its acute form and signs severity depends on the species, age, strain virulence and secondary infectious agents [11–14]*.* Including fever, respiratory signs, inappetence, marked depression, erosive stomatitis, catarrhal inflammation of ocular and nasal mucous, profuse diarrhea which may be watery, fetid and blood-stained, very often end-stage bronchopneumonia due to bacterial complications related to immunosuppression.

PPR virus is present in all kind of secretions. Animal excretions starting from 3 to 22 days post infection [15–17]. PPRV is usually transmitted via direct contact with infected animals, or through their fresh secretions or feces. Goats are considered more susceptible than sheep and occasionally wild small ruminants [18–22].

The World Organization for Animal Health (OIE) and The Food and Agriculture Organization of the United Nations (FAO) have set up a global eradication program based on epidemiological surveillance, early case finding and comprehensive vaccination campaigns.

In fact, deep knowledge of PPR disease and their clinical sign in target species it’s a fundamental basis of effective surveillance.

The current understanding of PPRV pathology has been assumed from the closely related rinderpest virus and other morbillivirus infections [23]. Very few studies focused on the pathology of PPRV has been performed and little is known about the mechanisms underlying establishment of the disease, pathogenesis, in susceptible animals.

In this study, experimental infection in two groups of goats with PPR MOR15, belonging to lineage IV, isolated locally in 2015 was performed and the resulting pathogenesis was evaluated using real time RT-PCR [24]. Moreover, we have followed disease signs and virus secretion, lesions and viral load in different tissues using a quantitative time-course study. We evaluate the pathogenesis of PPR genotype IV virus after infection of two groups of goats with viral load detection in secretions and tissues, compared to two uninfected goats. The aim of this study is to understand infection chronology, virus circulation, and contribute to the disease early detection.

## Results

### Hyperthermia

**Fig. 1:**
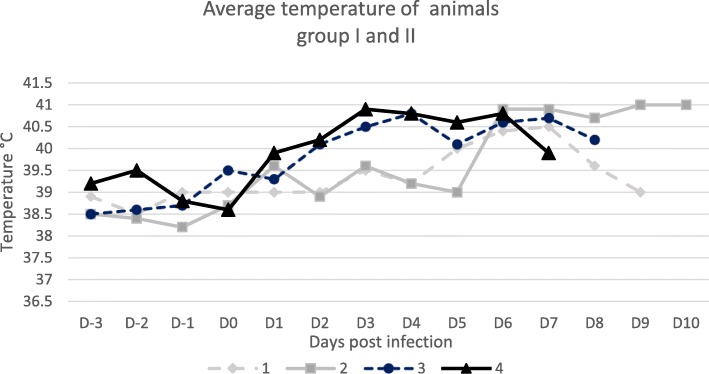
Rectal temperatures of goats following PPRV infection. Rectal temperatures were measured 3 days prior to experimental infection with PPRV (MOR15), and following infection every day until 9 dpi. Results presented are average temperature of four goats infected with cell virus suspension and infectious mashed tissue

Goats were allowed to acclimate to the laboratory environment for a quarantine period prior to experimental infection with PPRV. During that time, all experimental animals were healthy. First group of two goats 1 and 2 injected with viral suspension developed hyperthermia for 7 days, a peak was noticed at 7-day post infection at 40.7 °C with 3 and 5 days up to 40 °C (Fig. 1). The two goats 3 and 4 of group II (tissue virus) developed hyperthermia for 8 days, a peak at D4 at 40.9 °C; 7 and 6 days up to 40 °C for each goat. Goats of both groups showed a long hyperthermia period (above 39 °C) during 6 to 9 days. Group II presented an earlier hyperthermia and higher temperature values than group I (Fig. 1). The body temperature of two goats of group III 5 and 6 used as unvaccinated controls, remained normal and do not exceed 39,4 °C. Hyperthermia duration criteria was calculated by number of day > 39 °C.

### Clinical scoring

**Fig. 2:**
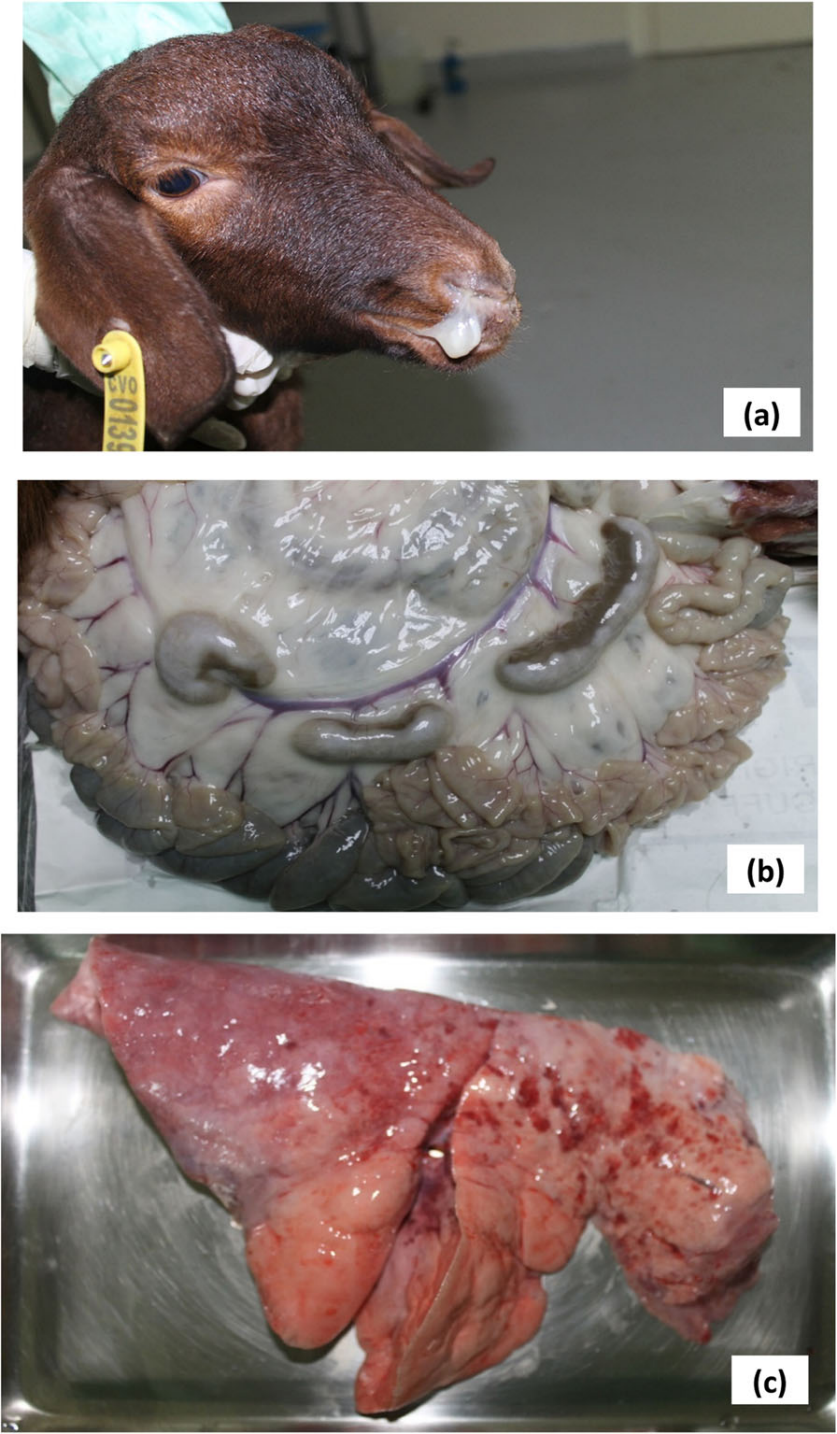
signs and lesions observed on goats. Nasal discharge (**a**), hypertrophy of mesenteric nodes (**b**) and lung congestion (**c**) observed on Peste des Petits Ruminants infected goat

Following inoculation with PPRV, four goats of the two groups showed typical PPR signs from 4 dpi, i.e. nasal and lacrimal discharges, coughing and dyspnea (Fig. 2). The two goats 1 and 2 of group I were euthanized at D9 and D10, presenting respectively a clinical score of 13 and 15. Both presented lacrimal, mucopurulent nasal discharges, coughing, diarrhea and asthenia. One goat presented a mild dyspnea and alimentation decrease in the last days. One goat of group II died at D9 with a clinical score of 14. The second goat was euthanized at D7 with a clinical score of 17. Both presented lacrimal, mucopurulent nasal discharges, dyspnea, coughing, diarrhea and asthenia. One goat presented an important dyspnea, depressive comportment and alimentation decrease in the last days (Table 1). The two goats of control group remained in healthy condition without any clinical sign and were euthanized at D10.

**Table 1:** Results of PPR experimental infection on goats according five severity criteria

Group	Animal	Mortality score	Clinical score	Tissue virus load score	Hyperthermia duration (days)	Swabs virus load score	Severity score
I	1	2	13	2.2	6	1.6	82.2
	2	1	15	2.8	8	2	91.4
II	3	2	14	2.7	9	1.9	94
	4	3	17	3.7	7	2.3	110.4

Group I was infected by cell virus suspension with a titer of 6.2 logTCID50/ml. Group II was infected by infectious mashed tissue (lung and mesenteric node). both from the same isolate. For each individual goat. a severity score was calculated. We evaluated five criteria by significance: 1) Mortality. 2); Clinical scoring; 3) Viral load in post mortem tissue; 4) Hyperthermia duration; 5) Viral load in swabs (number of positive and cycle threshold (Ct) values). Each criteria has a coefficient of significance (5 point for criteria 1 to 1 point for criteria 5). The severity score is a cumulative value of each criteria score. Previously multiplied by the coefficient of significance

### Viral excretion, viraemia and antibody detection

For both groups I and II lacrimal PPRV genome excretion was detected with similar value from D3, nasal PPRV genome excretion from D6, rectal PPRV genome excretion at D6. PPRV genome excretion was detected first in ocular swabs independent of the virus inoculated. Highest PPRV genome excretion value was obtained in rectal swabs followed by nasal swabs and lacrimal swabs. Viraemia began for group I at D6 with 40.4 Ct value respectively, for group II at D3 with 35.2 Ct value. Viraemia was higher and detected earlier in group II (Table 2). PPRV genome was not detected by PCR for all samples collected swabs and viremia of two goats of group III.

**Table 2:** individual Ct values per animal

		Days					
Samples	Animal Number	D0	D3	D6	D7	D9	D10
Nasal swabs	1	N	N	34,11		38,63	
	2	N	N	32,97		30,86	
	3	N	N	31,44	34,34	30,86	
	4	N	N	33,6		26,66	
Rectal swabs	1	N	N	28,43		26,21	
	2	N	N	32,33		22,35	
	3	N	33,86	25,28	24,57		
	4	N	N	31,82		20,09	
Lacrimal swabs	1	N	33,74	30,62		36,09	
	2	N	43,47	29,43		27,7	
	3	N	36,27	28,87	26,89		
	4	N	N	34,13		28,21	
Viremia	1	N	N	40,36		N	
	2	N	N	N		N	40,97
	3	N	35,16	35,59		36,39	
	4	N	N	38,55			

Real time RT-PCR threshold cycle (Ct) values in blood and swabs taken from 4 experimentally infected goats of group I and II (goats 1 to 4). Samples were collected for goat number 3 of group II euthanized at D7 and goat number 2 of group I was euthanized at D10. N: negative

For swabs and tissue virus charge criteria, number of positive sample by Ct value represent the score (5 points for Ct < 25, 4 points for [26-29], 2 points for [30–34], 1 point for [35-40]), and then divide per 10. The obtained swabs virus charge score for group I and II was respectively 1.8 and 2.1 (Table 1).

Consistent results were obtained for serological testing by VNT and bELISA. Both goats of group I had a 1.5 log10 value of antibody neutralizing titer at 9 dpi by VNT. The first detection of antibody seroconversion were detected from 6 dpi for one animal of group II with a 1.02 log10 value of antibody neutralizing titer by VNT. The second animal of group II died at D9 before we could take a sample for analysis. Animals evaluated positive by VNT were confirmed by bELISA. Goats of unvaccinated group remain seronegative for both VNT and bELISA.

### Post mortem lesion and viral detection in tissue

Prior to euthanasia, animals were anesthetized by intravenous administration of xylazine (Rompun 2%, Bayer) and intramuscular administration of ketamine (Imalgene 1000, Merial). Animals were partially exsanguinated by jugular catheterization.

At necropsy goats 3 and 4 of group II presented lesions of respiratory tract. The lungs were red with area firm to touch, mainly in the anterior and cardiac lobes (Fig. 2), trachea was congested and mesenteric and pulmonary nodes were enlarged and hypertrophied. Goat 3 presented inflammation of the pharynx with bacterial complication and petechial leaflet in lung. in the digestive tract he intestinal mucosa of the small intestine were moderately congested. For goats 1 and 2 of group I we observed congestion and hepatization lung, hypertrophy of mesenteric and pulmonary nodes, and one goat presented mild inflammation of intestine (Fig. 2). No lesions were observed at necropsy of two goats of unvaccinated group.

In both groups I and II, lung is the tissue with the higher viral detected charge (Ct value of 22.6 for group II and 27.1 for group I), followed by mesenteric, sub-maxillary and pulmonary nodes and abomasum with respectively average Ct values of 26.1, 27.8, 27.8, 28.0. In the spleen, intestine, liver, kidney and heart virus charge Ct values were 30.3, 31.2, 32.6, 35.6 and 37.4 respectively. The obtained tissue virus charge score for group I and II was respectively 2.5 and 3.2 (Table 1). Ct values in the organs of the two controls goats were negative.

For mortality criteria, the first to die at D7 had 3 points, the last one at D10, the two others had 2 point as they died at D9, Group II presented a higher mortality score (2.5) comparatively with group I (1.5) (Table 1).

Regarding the five selected criteria, goats 3 and 4 of group II presented a higher severity score (102.2) comparatively with of group I (86.8) (Table 1).

## Discussion

In spite of the importance of PPR as the priority disease for eradication, very few studies have been dedicated to describe infection kinetic in the animal body [20, 25–28]. The pathogenesis of PPR is still poorly understood and PPRV strains have different pathogenic profiles [15, 19]. it’s then important to properly understand the pathogenesis of the disease of different strains.

In our study, we demonstrated that experimental infection could reproduce typical PPR signs in young susceptible goats using a virulent strain, administered by the natural route of infection IN route associated with IV route.

Age and species are the key factor to succeed a PPR experimental infection. As described by several authors, susceptibility to the virus decrease rapidly with age. Adult animals can carry the virus with mild or no signs, which represent a constraint for diagnostic and surveillance. It is also known that goats are more sensitive than sheep especially for North African breed as described by many authors [19, 29, 30]. Alpine goats were used for a challenge model by Elharrak et al. (2012) with great success. However, in this study local goat breed showed similar sensitivity to PPRV as Alpine goats and could be used for challenge model.

We selected a virulent strain recently isolated in Morocco and tested on susceptible Alpine goats which showed a high clinical score with mortality [24]. It is a genotype IV virus, the most dominant in the world originated from Asia. We confirm in our study the high virulence of the used 2015 Morocco strain (mortality up to D10) comparatively to the 2008 Morocco strain for which mortality occurs later (up to D33) [31]. West African dwarf goats inoculated by culture of PPRV belonging to lineage I died from D28, with clinical signs of pyrexia, serous-mucopurulent nasal discharges with copious ocular discharge, sneezing and coughing, while oral erosions, profuse diarrhoea after the first 15 dpi [29]. In comparison with other strains from lineages II and IV, oral lesions were seen at 5–9 dpi, diarrhoea at 8–10 dpi and death 7–12 dpi [19]. 86% of hill goats (13 of 15) experimentally infected with a virulent Indian isolate (PPR/Izatnagar/94) died between 9 and 13 days pi [25].

Used route of infection is the most appropriate since IN spray reproduce the natural infection conditions and the IV injection allow to secure a minimal quantity of the virus administered.

To appreciate the severity of the infection we considered the five criteria listed in the results chapter. The early mortality observed in all animals was associated with a high clinical score. Similar signs has been also observed by several authors confirming that PPR is dominated by pulmonary signs [20, 31, 32]. With our strain we did not observed any lesions at buccal mucosa.

PCR analysis of collected swabs suggest that animals became contagious from D3 through lacrimal secretion then nasal secretion and faeces from D6. Rectal swabs are the most indicated for diagnostic and epidemio-surveillance with the highest Ct value detected. Nasal and lacrimal secretion represent the first and the direct way of virus transmission.

Increasing body temperature was reported in group I at D5 pi with a maximum at D7pi while in group II hyperthermia started at D2 at to D7 pi. The temperature reached 40.5 °C at D7 and D4 for group I and 2 respectively. Despite the hyperthermia, very low viraemia is detected by PCR.

In the study, characteristics lesions of PPR disease were obtained in post mortem tissues. Lung is the tissue with the highest viral detected charge (Ct 22.6 for Group II and 27.1 for Group I). followed by mesenteric, sub-maxillary and pulmonary nodes and abomasum (with average Ct value of 27.5). Lower Ct were obtained in the spleen, intestine, liver, kidney and heart (30.3, 31.2, 32.6, 35.6 and 37.4 respectively). Lung is the preferred source of virus among all other tissues for virus detection and isolation.

Signs, lesions, mortality and viral charge are clearly expressed in group II (infected with tissue mixture) comparatively to group I (infected by cell suspension). This difference can be attributed to the virus titre even if it can not be determined for the group 2, while with cell culture the used titre (6.5 log TCID50/ml) seems to be enough to reproduce a typical disease. Cell culture is always preferable to tissue because of purity concern and known titre. However. in our study, the use of infected tissue is much more efficient than the cell culture suspension virus probably because of virus adaptation on cells.

## Conclusions

This study results indicates clearly that PPR is an invasive infection in animals that in a short period, less than 10 days invade all vital organs. Transmission to naïve animals may starts 2 days after infection and last for certainly more than 2 weeks as in this study animals died quickly. Swabs and tissues showed high charge of the virus and clinical signs dominated by pulmonary attack and digestive symptoms secondary. On live animals early diagnostic may be easily done on lacrimal swabs, rectal swabs show the lowest Ct. Virus isolation could be done on swabs or lung tissue. Antibody seroconversion could be detected beginning from 6 dpi.

The vaccine efficacy test on animals consist of vaccination, followed by experimental infection of vaccinated and unvaccinated animals by using virulent strain of PPRV. The vaccinated animals should be completely protected after the experimental infection and unvaccinated animals must show typical signs of PPR. The obtained results demonstrate that the 2015 Morocco isolate, genotype IV, is highly virulent and could be used in challenge to test vaccine efficacy under specified conditions.

## Methods

### Animals

Infection was carried out according to international guidelines described for the care and handling of experimental animals, chapter 7.8 of the Terrestrial Animal Health Code and Directive 2010/63/UE of the European commission [32, 33]. The protocol was submitted and approved by the internal Laboratory Committee.

Six local breed goats, males, aged 6 to 9 months, of around 30 Kg, are arranged to three groups of 2 goats each. Animals were randomly chosen from MCI Santé Animale breeding farm located at Sidi Yahya Zaer in North-West region of Morocco Animals. Goats were fed a complete balanced diet and water ad libitum and housed in animal boxes (Biosecurity level 3 containment) at MCI Santé Animale.

Animals were maintained 2 weeks under observations before starting the following experiments. Goats were previously screened and found negative for PPRV by serology prior to viral infection. Animals were tested negative for PPRV specific antibody by ELISA using the blocking enzyme-linked immunosorbent assay (bELISA) and virus neutralization test (VNT).

Group I (goats 1 and 2) was infected by cell virus suspension and group II (goats 3 and 4) by infectious mashed tissue (lung and mesenteric node), both from the same isolate. Group III (goats 5 and 6) was inoculated by placebo solution and this group was in a separated room from the other groups.

### Virus strain

The PPR Moroccan virulent strain MOR15 strain used for the experiment was isolated at MCI Santé Animale laboratories, during the 2015 outbreak in Morocco from a lamb showing characteristic clinical signs of PPR and it belonging to lineage IV [24].

The cell virus suspension had a titer of 6.2 logTCID50/ml. The viral stock was passaged 8 times in Vero (African green monkey kidney cells, ATCC No.CCL-81). Infected cells and supernatant were harvested and frozen at − 80 °C for subsequent experiments. Virus titration was performed using the method described in the OIE Terrestrial Manual (Chapter 2.7.11).

The infectious mashed tissue was prepared from lung and mesenteric node presenting a Ct of 20.2 in qPCR.

### Experimental infection, animal sampling and monitoring

The experiment infection was carried out according to the protocol described by El Harrak et al. (2012) [34]. Each goat of the two inoculated groups was infected by two different routes of inoculation, 1 ml via intravenous (IV) route and 1 ml via intra-nasal (IN) spray.

All animals were observed daily, with rectal temperature, clinical signs and clinical scoring recorded throughout the study. The rectal temperature was recorded for each animal of each group during the quarantine period by repeated measurements. If a rectal temperature exceeds above 39 °C, we defined Hyperthermia.

A clinical scoring was applied with notation system from 0 to 4 based on the gravity of different clinical symptoms observed on goats during the experiment: general clinical appearance, hyperthermia, alimentation, behavior, diarrhea, nasal discharge, salivation, respiratory signs including dyspnoea, coughing and sneezing. These signs were scored as described in Table 3. Lacrimal, nasal and rectal swabs, as well as blood samples were collected from goats every 3 days post infection (dpi) and analysed by quantitative real-time reverse transcriptase-polymerase chain reaction (RT-qPCR) to monitor viral load.

**Table 3:** Scoring of recorded clinical signs

Clinical signs	Score
General behavior	
Normal	(0)
Inactif	(1)
Low head	(2)
Very inactif	(3)
Laying down	(4)
Food uptake	
Normal	(0)
Loss of appetite	(2)
Anorexia	(3)
Absence	(4)
Faeces	
Normal	(0)
Soft to liquid	(1)
Liquid diarrhea	(3)
Hemorrhagic diarrhea	(4)
Nasal secretions	
Normal	(0)
Liquid	(1)
Serous	(3)
Muco-purulent	(4)
Hyperthermia	
Normal	(0)
40 < t°C < 40.5	(2)
40.5 < t°C < 41	(3)
t°C > 41	(4)
Cough	
Normal	(0)
Moderate	(2)
Frequent and dry	(3)
Frequent and productive	(4)
Salivation	
Normal	(0)
Moderate	(2)
Profuse	(3)
Respiration	
Normal	(0)
Moderate	(2)
Marked	(3)
Marked with difficulty	(4)

Clinical signs recorded of all animals: general behaviour, food uptake, faeces, nasal secretions, hyperthermia, salivation, cough, and respiration

If severe clinical symptoms were observed and the cumulative clinical score arrived at a value between 15 and 18, goats were euthanized by overdose anesthesia through intravenous xylazine and intramuscular ketamine injections followed by exsanguination [34, 35].

Post mortem samples were collected from the lungs, abomasum, liver, spleen, heart, kidney, intestine, mesenteric, sub-maxillary and pulmonary lymph nodes.

For each individual goat, a severity score was calculated. We listed five criteria by significance: 1) Mortality, 2); Clinical scoring; 3) Viral load in post mortem tissue; 4) Hyperthermia duration; 5) Viral load in swabs (number of positive and cycle threshold (Ct) values). Each criteria has a coefficient of significance (5 point for criteria 1, 4 point for criteria 2, 3 point for criteria 3, 2 point for criteria 4 and 1 point for criteria 5). The severity score is a cumulative value of each criteria score, previously multiplied by the coefficient of significance.

### Sample treatment

All biological samples were transported to the laboratory on ice and analyzed by PCR for viral genome detection. Swabs were collected in 2 ml PBS supplemented and centrifuged at 2000 rpm for 20 min at 4 °C. Tissues were homogenized and centrifuged at 2000 rpm for 20 min at 4 °C. The supernatant was kept and used for analysis. The blood samples were directly used for RNA extraction.

### Samples analysis

Animals were tested negative for PPRV specific antibody by virus neutralization test (VNT) and ELISA using the blocking enzyme-linked immunosorbent assay (bELISA). Serological testing was carried out every 3 days.

VNT test was done as described in the OIE Terrestrial Manual [13]. The test was based on serial 1:3 dilutions of heat inactivated sera, mixed with a constant dose of PPR virus (100 TCID50), incubated for 1 h, and then inoculated onto Vero cells to be observed for neutralization of cytopathic effect (CPE) after 7 days of the incubation period at 37 °C with 5% of CO2. The neutralizing antibody titer was calculated in accordance with Reed and Muench method.

bELISA was developed, tested and validated by the Pan African Veterinary Vaccine Centre of the African Union (AU-PANVAC) [36]. Sera samples from goats were tested at a 1:100 dilution for PPRV antibody by an ELISA using PANVAC PPR-Ab check bELISA purchased from AU-PANVAC. Briefly, 25 μl of sera samples were added to 96 and incubated for 1 h at 37 °C. After washing the wells of the plates with PBS, 100 μl of an Monoclonal antibody C4F3 & conjugate anti-mouse was added to fix the remaining free epitopes. After 45 min of incubation 37 °C, 100 μl of 3,30,5,50-Tetramethylbenzidine (TMB) substrate solution were added to each well. The reaction was stopped after 15 min at room temperature by the addition of 100 μl of 0.16 M sulfuric acid, and then the reactivity results were read at 450 nm. Antibody positive and negative cut-off values were calculated as recommended by manufacturer with the sera being negative, if a percentage of inhibition was < 30%, and positive if a percentage of inhibition was > 30%.

All the samples were screened for viral genome detection by qRT-PCR. RNA extraction was carried out using an RNA extraction kit (Bioline BIO-52075, isolate II RNA Mini kit). Real time RT-PCR amplification and detection was performed using a Applied Biosystem 7500 real time PCR system with the SensiFast Probe Lo Rox one step kit (Bioline). Viral genome detection was performed using primer and probe described by Batten [37]. specific for nucleocapsid (N) gene. Briefly, each 25 μl reaction contained 4 μl extracted RNA; 12.5 μl of 2x SensiFAST Probe Lo-ROX One-Step Mix; 0.5 μl probe (10 μM); 1 μl (10 μM) forward and reverse primer and 6 μl nuclease free water. The cycling conditions were as follows: Reverse transcription 45 °C for 10 min; reverse transcription inactivation and DNA polymerase activation 95 °C for 2 min; followed by 40 cycles of amplification (95 °C for 5 s and 60 °C for 20 s).

## Data Availability

The datasets used and/or analyzed during the current study available from the corresponding author on reasonable request.

## Abbreviations

BSL3: Biosafety level 3
CT: Cycle Threshold
DPI: Day post infection
ELISA: Enzyme-linked immunosorbent assay
FAO: Food and Agriculture Organization
IN: Intra-nasal
IV: Intravenous
OIE: World Organization for Animal Health
PPRV: Peste des petits ruminants virus
RT qPCR: Real-time reverse transcriptase-polymerase chain reaction
VNT: Virus neutralization test
